# The Promise of Stochastic Resonance in Falls Prevention

**DOI:** 10.3389/fphys.2018.01865

**Published:** 2019-01-28

**Authors:** Olivier White, Jan Babič, Carlos Trenado, Leif Johannsen, Nandu Goswami

**Affiliations:** ^1^INSERM UMR1093-CAPS, Université Bourgogne Franche-Comté, UFR des Sciences du Sport, Dijon, France; ^2^Acquired Brain Injury Rehabilitation, Faculty of Medicine and Health Sciences, School of Health Sciences, University of East Anglia, Norwich Research Park, Norwich, United Kingdom; ^3^Laboratory for Neuromechanics and Biorobotics, Jožef Stefan Institute, Ljubljana, Slovenia; ^4^Leibniz Research Centre for Working Environment and Human Factors TU Dortmund (ifADO), Institute of Clinical Neuroscience and Medical Psychology, University Hospital Düsseldorf, Düsseldorf, Germany; ^5^Otto Loewi Research Center for Vascular Biology, Immunology and Inflammation, Medical University of Graz, Graz, Austria

**Keywords:** stochastic resonance, balance disorder, orthostatic intolerance, aging, falls

## Abstract

Multisensory integration is essential for maintenance of motor and cognitive abilities, thereby ensuring normal function and personal autonomy. Balance control is challenged during senescence or in motor disorders, leading to potential falls. Increased uncertainty in sensory signals is caused by a number of factors including noise, defined as a random and persistent disturbance that reduces the clarity of information. Counter-intuitively, noise can be beneficial in some conditions. Stochastic resonance is a mechanism whereby a particular level of noise actually enhances the response of non-linear systems to weak sensory signals. Here we review the effects of stochastic resonance on sensory modalities and systems directly involved in balance control. We highlight its potential for improving sensorimotor performance as well as cognitive and autonomic functions. These promising results demonstrate that stochastic resonance represents a flexible and non-invasive technique that can be applied to different modalities simultaneously. Finally we point out its benefits for a variety of scenarios including in ambulant elderly, skilled movements, sports and to patients with sensorimotor or autonomic dysfunctions.

## The Challenge of Healthy Aging

Life expectancy is increasing globally and functional contributions to society post-75 years are highly attainable. Over one third of adults over the age of 65 fall each year (Sattin et al., 1990). Two million patients present annually to emergency units, worldwide, complaining of dizziness upon standing up or after an accidental fall (Goswami, 2017; Goswami et al., 2017). Falls are ranked within top three causes in terms of years lived with disability in most regions of the world (WHO). Falls are not only associated with injury and morbidity, but also to reductions in physical, psychological, and social capacities (Myers et al., 1996; Blain et al., 2016; Bousquet et al., 2017). The direct cost of falling exceeds $10 billion a year in the United States, with almost 9,500 deaths per year attributed to falling alone (Myers et al., 1996). The total costs of falls in the population of the United Kingdom has been estimated from more than two billion GBP to up to 4.4 billion GBP per year by the NHS (Public Health England with the National Falls Prevention Coordination Group Member Organisations, 2017) and is predicted to rise further. Critically, the consequences of a fall after 75 years old are much worse than between 65 and 75 years old and include fracture, frequent hospitalizations, increased morbidity and mortality. Preventing the occurrence of such events is therefore of paramount importance.

Epidemiological studies have shown that 30–70% of falls occur while walking on level ground. However, simple it appears, bipedal ambulation is demanding in many ways and gait relies on complex sensorimotor integration. The act of balancing on our lower extremities is not learned until about 10–12 months of age. A healthy sensory system is necessary for successful postural control and locomotion. Indeed, the central nervous system must maintain accurate estimates of the position of the body in space and of the limbs in relation to each other (proprioception). Efficient postural reflexes must be triggered when external perturbations are detected. Furthermore, appropriate cardiovascular responses that keep blood pressure stable after the transition from lying/sitting to standing are required. The act of balancing needs to become automatic so that other tasks will not jeopardize it by interfering. During senescence, impairments in one or more of these systems, including cognitive functions, lead to increased risk of falls. Falls in elderly and impaired individuals are a complicated phenomenon comprising multi-factorial intrinsic and extrinsic risks (Shumway-Cook et al., 1997). Intrinsic factors, or those related to the individual, include a decreased performance in the balance control system, with loss of mobility being a strong indicator for increased fall risk. In order to maintain stability, adequate levels of vision, vestibular function, musculoskeletal function, and proprioception are all required. Extrinsic factors, or those pertaining to environmental hazards, contribute significantly to fall incidents and include obstacles to trip over, poor lighting, slippery surfaces, or inappropriate furniture. Hence, an in-depth understanding of the multitude of neuromuscular, cognitive, sensory, sociological and environmental factors that contribute to balance control are necessary for early diagnosis and treatment of elderly who present significant risks of falls.

Since the process of balance control relies on many physiological factors and sensory signals, maintaining the quality of these signals at their optimal level is fundamental. Here, we review how a highly promising phenomenon called *stochastic resonance* could play an important role toward addressing balance control, and consequently, toward falls prevention.

## Stochastic Resonance: When Noise Becomes an Allied to the Brain

To ensure optimal control of a system, adequate and accurate information inputs are required. For instance, navigating a computer mouse to a button on the screen entails the estimation of target position but also the hand/mouse position. These are available only through (biological) sensors that also embed uncertainties and are sometimes available as very weak delayed signals. Stochastic resonance can help enhancing detection and processing of a weak signal blurred by the many sources of uncertainties and perturbations.

### The Concept of Stochastic Resonance

**FIGURE 1:**
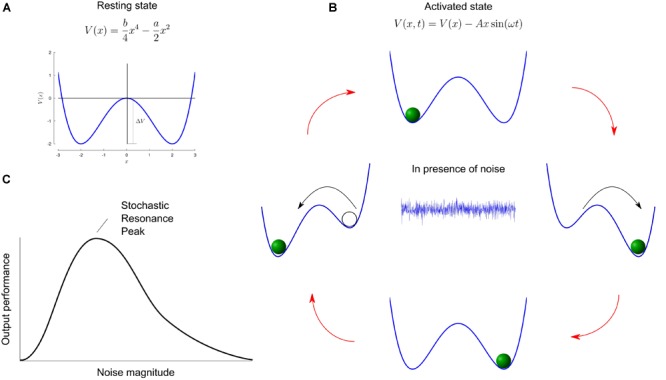
Mechanism of stochastic resonance. **(A)** Sketch of a double well potential V(x). In this example, the values a and b are set to 2 and 0.5, respectively. The minima are located at $x=\pm \sqrt{\frac{a}{b}}$ and are separated by a barrier potential $\Delta V=\frac{a^{2}}{4b}$. **(B)** In the presence of periodic driving, the height of the potential barrier oscillates through an antiphase lowering and raising of the wells. The cyclic variations are depicted in the cartoon. A suitable dose of noise (represented by the central white noise plot) will allow the marble to hop to the globally stable state. **(C)** Typical curve of output performance versus input noise magnitude, for systems capable of stochastic resonance. For small and large noise, the performance metric is very small, while some intermediate non-zero noise level provides optimal performance. Panels **A,B** adapted from Gammaitoni et al. (1998).

In linear systems, optimal performance is obtained in the absence of noise. However, restricting models of natural systems to noise-free and linear systems is not realistic. First, noise is ubiquitous and can never be completely eliminated. Second, systems with thresholds characterize a large class of non-linear systems, e.g., from neurons to behaviors (such as the perception of sensory stimuli). This is where the concept of stochastic resonance becomes interesting: it describes any phenomenon where the presence of noise in a non-linear system improves the quality of an output signal compared to when there is no noise (McDonnell and Ward, 2011). Intuitively, noise is usually thought of as detrimental and associated with words such as nuisance or undesirable, and the concept of it being useful is apparently contradictory. The rationale behind stochastic resonance is concisely illustrated in the work of Gammaitoni et al. (1998). Let us consider a marble moving in a symmetric double well potential V(x) (Figure 1A). Without any external actuation force, the mass remains stuck in one of the wells. A potential barrier (ΔV) prevents the marble from swapping positions between wells. When a small periodic driving force is submitted to the system, it generates oscillations of the deepness of each well. Still, the potential barrier – although decreased compared to its resting state – is too high too allow transitions. An appropriate small dose of noise (*stochastic*) injected into the system will constructively (*resonate*) combine with the driving force and statistically allow periodic transitions between the wells. Hence, adding a small noise to a weak input signal can make the resultant signal surpass the threshold of a given neuron or neural circuit and hence provide useful information about the input weak signal for the central nervous system. On the other hand adding a too large unrelated noise signal will make the output of the threshold detection non-linear system useless in terms of providing information about the weak input signal. A trade-off must then be sought between these two extremes (Figure 1B). The graphical representation of such trade-off, namely a U-like curve represents the signature of the stochastic resonance phenomenon (Figure 1C).

By adopting this concept of stochastic resonance, it becomes possible to detect a weak signal using noise. In that case, the signal exceeds the detection threshold stochastically, generating an information that is transmitted to the output. This concept has been successfully demonstrated and applied to optimize logic operations in genetic regulatory networks blurred by various forms of noise (Wang and Song, 2016; Zhang and Song, 2018). The presence of a control noise in terms of a forcing signal enhanced the reliability of the logical function implemented in those networks. Furthermore, stochastic resonance is also effective to detect subthreshold signals at the single neuron level and can even be optimized in neuronal networks (Chen et al., 2008). Interestingly, this phenomenon does not only enhance the detection of subthreshold – but also superthreshold – signals in feedforward neuronal networks (Stocks, 2000; Yu et al., 2016). In biological systems, a threshold is reached when biological sensors (cutaneous mechanoreceptors in hands or feet, proprioceptors in muscles and joints, hair cells in the inner ear, etc) receive a signal. However, the amplitude of that signal must be large enough to elicit a response to the central nervous system that will eventually, once processed by the brain, trigger an action (e.g., a postural adjustment to prevent a fall). Unfortunately, these thresholds increase with age (Wells et al., 2003) hampering their detection. Stochastic resonance therefore makes the signal detectable again, as if this technique actively adapted the sensitivity of the sub-optimal sensor. In that way, the system can maintain the same responsiveness to hazardous situations. Therefore, shortening reaction latencies through decreased information processing time puts a system in better conditions to circumvent unwanted effects, such as falls (Toledo et al., 2017).

Stochastic resonance can be applied to a range of physiological systems. This technique has been shown to improve detection of low tactile stimuli in the hand mechanoreceptors (Collins et al., 2003; Moss, 2004; Stein et al., 2005; Trenado et al., 2014c) and various motor functions (Richardson et al., 1998; Kitajo et al., 2003; Aihara et al., 2008; Mulavara et al., 2011; Trenado et al., 2014a). Furthermore, noisy (stochastic) stimulation of the vestibular system has the potential to improve motor functions (Pan et al., 2008; Samoudi et al., 2014; Lee et al., 2015), postural stability (Pavlik et al., 1999; Pal et al., 2009; Mulavara et al., 2011; Samoudi et al., 2014; Inukai et al., 2018), may prevent orthostatic intolerance (symptoms when standing upright) and cardiovascular responses (Soma et al., 2003; Yamamoto et al., 2005; Tanaka et al., 2012), and possibly also auments cognitive functions (Yamamoto et al., 2005; Pan et al., 2008; Wilkinson et al., 2008; Kim et al., 2013). Since stochastic resonance emerges in any threshold-activated system, its effects may be relatively independent of any underlying pathology affecting perceptual uncertainty in sensory systems. All the above mentioned physiological systems are relevant for maintaining a stable body balance. While the effect of stochastic resonance may be small in absolute terms, utilizing it in situations where margins are important can lead to large benefits. The principle of system enhancement by stochastic resonance is well documented, and several small studies indicated improvements in more than one domain of balance control during exposure to sensory noise via different sensory modalities (multisensory stochastic resonance). However, this promising technique has thus far only been tested in a limited number of patients and healthy controls and for durations not exceeding 24 h. In the following sections, we review in detail these effects and identify emerging applications.

### The Vestibular System

The technique of applying stochastic vestibular stimulation instead of using a more traditional square-wave or sum of sinewave signals is relatively new. Several studies have focused on performance improvement with stochastic resonance applied on the vestibular system such as body responses in posture, balance, and gait (Fitzpatrick et al., 1996; Pavlik et al., 1999; Scinicariello et al., 2002). In addition, stochastic vestibular stimulation at imperceptible levels improves stability during balance tasks in normal, healthy subjects (Mulavara et al., 2011, 2012). Similarly, these stimulations also improve ocular stabilization reflexes in response to whole-body tilt and postural balance performance on an unstable compliant surface in Parkinsonian patients (Pal et al., 2009; Samoudi et al., 2014).

The vestibular system is connected to spinal, cerebellar and cerebral motor control structures and can be selectively activated with external electrodes. A constant current stimulator developed to ensure imperceptibility of electrical stimulation of the vestibular system by subjects has been engineered (Mulavara et al., 2011). A series of evaluations on the efficacy of stochastic vestibular stimulation during standing and walking on unstable surfaces and on perception of tilt sensation in otolith-canal (intra-vestibular) conflict scenarios has also been done (Wuehr et al., 2016a,b). These researchers have evaluated the frequency characteristics of the electrical stimulus to optimize balance performance of subjects standing on a compliant surface with their eyes closed. Low imperceptible amplitude bipolar binaural electrical stimulation of the vestibular organs was applied using a constant-current stimulator through electrodes placed on the mastoid processes in the range of 30–330 μA root mean square (RMS) in 30 μA RMS steps. Using measures of head and trunk stability and a multivariate optimization criterion, and by employing a white noise-based stochastic stimulation signal, these researchers have shown that the stochastic vestibular stimulation in the range of 30–120 μA RMS improved balance performance in normal healthy subjects as well as in patients with Parkinson’s disease. More recently, it was also shown that noisy galvanic vestibular stimulation can enhance roll vestibular motion perception (Keywan et al., 2018). Additionally, cross-planar improvements in balance performances in the range of 5–26% in normal healthy control subjects have been seen. These results indicate that stochastic vestibular resonance may be sufficient to provide a comprehensive countermeasure approach for improving postural stability.

### Haptics and Somatosensory Perception Are Relevant for Balance Control

For the control of body sway, human postural control system can flexibly utilize different sensory channels, such as non-plantar skin receptors. Uncertainty about the state of the body or a limb, such as its position in space can lead to erroneous motor planning and, consequently, injuries or falls. Should additional tactile and proprioceptive information become available, however, more accurate state estimates based on multisensory integration and prediction may decrease the likelihood of unsuccessful behaviors (Wolpert and Ghahramani, 2000; Todorov, 2004).

Mechanically non-supportive fingertip contact (<1 N) with an earth-fixed reference reduces body sway in quiet standing with eyes closed and may be even more efficient than vision alone (Holden et al., 1994; Jeka and Lackner, 1994; Lackner et al., 1999). A review by Baldan et al. (2014) on the effect of light touch on postural sway in individuals with balance problems due to aging, brain lesions or other motor or sensory deficits concluded that light touch leads to very reliable body sway reductions irrespective of the underlying balance impairment. Most studies demonstrated the benefits of haptic feedback exclusively in a quiet standing posture but improvements have also been reported in more dynamic postural activities, such as balance compensation following a mechanical perturbation (Dickstein and Laufer, 2004; Johannsen et al., 2007, 2017; Forero and Misiaszek, 2013), overground and treadmill walking (Dickstein and Laufer, 2004; Fung and Perez, 2011; Forero and Misiaszek, 2013; Kodesh et al., 2015) and staircase negotiation (Reeves et al., 2008; Reid et al., 2011).

Reductions in body sway during upright standing observed with light fingertip contact to an external stationary reference can be further reduced by the application of vibratory noise to the touch contact (displacement <0.1 mm) (Magalhães and Kohn, 2011). A typical experimental setting that can be used to investigate these aspects is discussed in detail in Magalhães and Kohn (2011). Briefly the participant’s posture is measured by means of a force plate. The area covered by the projection of the trajectory of the center of mass serves as performance index. Measurements can be performed in different sensory conditions involving the provision of visual feedback or not, and the availability of an external reference for fingertip light touch. Values obtained in experimental conditions are compared to a defined baseline performance. In the stochastic resonance condition, vibratory noise stimuli is applied to the fingertip. Power of low frequency components of body sway (<0.5 Hz) were particularly responsive to the vibratory noise stimulation (Magalhães and Kohn, 2011). This effect of vibratory noise stimulation on the frequency components of body sway, however, seems to depend on the relative vibratory amplitude. Kimura et al. demonstrated that vibratory stimulation with an amplitude of the 50% of each participant’s vibrotactile threshold caused reduction in the power of high-frequency components (>1 Hz) of body sway in contrast to greater stimulation amplitudes (Kimura et al., 2012).

Aging is accompanied by a constant decline of the sense of touch: between the age of 20 to 80 years, acuity thresholds increase by approximately 1% per annum with the fingertips tending to be one of the most vulnerable body parts (Wells et al., 2003). This loss of sensory acuity impacts on various aspects of function in the elderly, including the manual handling of objects and control of postural stability. These impairments reduce quality of life and independence because elderly lose dexterity and might have falls with serious physical injury. However, older adults retain the ability to use fingertip contact for augmentation of body sway feedback despite reductions in their tactile sensitivity (Tremblay et al., 2004). In fact, the efficacy of tactile feedback for sway reduction seems to be even greater in older compared to younger adult participants perhaps due to more severe aging-related loss of somatosensory information in the distal parts of the lower extremities (Baccini et al., 2007). Differential effects noted between young and elderly indicate that elderly people gain more in motor control performance than do young people with the application of noise to the feet (Priplata et al., 2003). The benefits of tactile information for the control of body balance in quiet stance as well as dynamic activities is robust and has been demonstrated in a number of neurological disorders.

### Body Balance

Increased postural instability and falls risk due to impaired somatosensation is associated with reduced plantar sensation in older adults, patients with diabetic neuropathy and stroke patients. In the past one and a half decades, however, the effect of stochastic resonance by noise enhanced stimulation on balance control has been demonstrated as a very robust phenomenon. For example, a meta-analysis by Woo et al. concluded that enhanced noise stimulation of the lower limbs results in moderate to high effects sizes on parameters of postural regulation and stability (Woo et al., 2017). Changes in postural performance with noise enhanced stimulation were evaluated with respect to diverse postural activities, such as standing and walking, the body location of the applied stimulation, such as the soles of the feet and more proximal regions of the lower extremities and the fingertips. In the majority, the stimulation modality was vibrotactile but electrical and auditory stimulation have also been used. Finally, target populations ranged from healthy young adults to patients with neurological impairments and reduced somatosensation. A systematic review by Bagherzadeh Cham et al. (2016) on the benefits of subthreshold vibratory noise stimulation of the soles of the feet in older adults and diabetic individuals concluded that vibratory stimulation improves balance and gait performance in these populations.

Mechanoreceptors in the soles of the feet, providing information about pressure distribution and shear forces, pose an important sensory modality for the control of posture during quiet standing as well as during walking. This is evidenced by systematic alterations of the center of pressure trajectories during quiet standing during suprathreshold vibrotactile stimulation of the plantar foot zones as well as plantar electrical stimulation in neurologically healthy individuals (Kavounoudias et al., 1998, 1999, 2001; Roll et al., 2002). These involuntary drifts away from the equilibrium state during suprathreshold vibrotactile stimulation may be a cause why suprathreshold vibrotactile noisy stimulation has a destabilizing effect on postural control in young and older adults (Simeonov et al., 2011).

In contrast to suprathreshold levels of vibratory stimulation, Priplata et al. demonstrated that subthreshold vibratory noise stimulation improves body sway stability in patients with peripheral and central somatosensory deficits such as following diabetic neuropathy and stroke (Priplata et al., 2006). This was also demonstrated for a more dynamic postural task such as Timed-Up-and-Go (Podsiadlo and Richardson, 1991), in which subthreshold (70–85% of the sensory threshold) plantar vibrotactile stimulation increased complexity of sway dynamics and mobility (Zhou et al., 2016). Not only performance in the Timed-Up-and-Go improves during plantar subthreshold vibrotactile stimulation but temporal gait variability is also reduced in older adults (Lipsitz et al., 2015). The individual falls risk seems not to play a role with respect to the benefit on gait variability (Galica et al., 2009). Stephen and coworkers showed, however, that the effect of noise-enhanced vibrotactile stimulation on gait variability in older adults is dependent on participants’ initial variability levels (Stephen et al., 2012). Individuals with relatively low initial gait variability showed increases, while for those who expressed high gait variability initially, variability was strongly reduced. This shows that not every individual will gain an advantage of subthreshold vibrotactle noise stimulation. The frequency and the amplitude of plantar vibrotactile stimulation is relevant, as these affect long-range correlations in stride length and stride interval during walking in healthy young adults (Chien et al., 2017).

Kelty-Stephen and Dixon (2013) reanalyzed the original data by Priplata et al. (2002) and found that the effects of subthreshold noisy vibrotactile stimulation on body sway dynamics are modulated by the degree of autocorrelations present in body sway, which could explain differences in interindividual responsiveness. It is therefore crucial to adjust the stimulation parameters to an individual’s specific requirements. For example, Wells et al. applied an adaptive psychophysical procedure to assess participants’ vibrotactile thresholds of the foot soles in younger and older adults and demonstrated that subthreshold vibratory noise stimulation increased their plantar sensitivity (Well et al., 2005). Further, they suggested that their procedure allows a reliable *a priori* determination of participants’ optimal stimulation noise level to augment plantar vibrotactile sensitivity.

Subthreshold vibrotactile noise stimulation augments postural performance under more difficult task conditions too. Noise-enhanced vibrotactile stimulation benefits gait variability in terms of stride width in a fatigued state, for example during inclined treadmill walking with 30% added body weight (Miranda et al., 2016). Dettmer et al. (2015) tested young and older adults in an intersensory conflict situation by sway-referencing the visual input, thereby removing any visual sway-related information. In this context, older adults have been shown to show dramatically increased postural instability due to overreliance on visual information for body sway control (Simoneau et al., 1999). Vibrotactile noisy stimulation was found to improve balance performance particularly in the older adults (Dettmer et al., 2015). This demonstrates the stimulation’s potential to attenuate age-related visual overreliance and susceptibility to intersensory conflict. However, in a follow-up study which increased the challenge of balance control during standing by the addition of a secondary working memory task, Dettmer et al. (2016) did not find an enhanced effect of vibration despite moderate correlations between the vibrotactile plantar sensitivity and postural parameters.

A limitation of the capacity of subthreshold vibrotactile noise stimulation to reduce body sway seems to exist, however, when the postural context becomes too complex. Keshner et al. (2014) challenged control of body balance with two kinds of disruptive stimuli alone and in combination in healthy young adults: a continuous optokinetic visual field rotation around the pitch axis, which imposed intersensory motion conflict, and a mental calculation task imposing additional attentional demands. While plantar subthreshold noisy vibrotactile stimulation reduced body sway during either mental calculation or visual stimulation, the increased task complexity of the visual-conflict-dual-task combination diminished the influence of subthreshold vibrotactile stimulation on the dynamics of body sway. Also, it is important to consider, that the sensitivity of the soles of the feet to vibrotactile stimulation can be affected by the general postural context (Mildren et al., 2016).

The alternative to subthreshold vibrotactile noisy stimulation of mechanoreceptors is electrical stimulation muscle proprioception. For example, subthreshold electrical noisy stimulation of the lower leg muscles (tibialis anterior, triceps surae) in seated and standing participants expressed a connection between reduced variability in isometric plantar flexion force and reduced body sway (Magalhães and Kohn, 2012, 2014). Cutaneous receptors respond to electrical noise stimulation as, when applied at the knee, it reduces sway during single-leg standing in older adults (Gravelle et al., 2002). Finally, in terms of balance control, auditory white noise stimulation reduces the variability in both the lower and higher frequency components of postural sway in young and older adults as well (Ross and Balasubramaniam, 2015; Ross et al., 2016).

### Hand Function and Tactile Sensitivity

As mentioned above, subthreshold vibrotactile noise stimulation to the fingertips augments balance performance. It also improves performance in manual fine motor control. For example, Sueda et al. (2013) demonstrated that subthreshold vibratory noise applied to the grip of a surgical device improves the tactile sensitivity mediated by the device. Similar effects were reported by Sawada et al. (2015). Furthermore, Beceren et al. (2013) demonstrated that adjusted subthreshold vibratory noise to the fingertips reduces the sensitivity threshold. Interestingly, normal vibration of the fingertip differed from the tangential vibration in their study. While normal vibration seemed to stimulate mainly fast-adapting Type I fibers, the tangential vibration also resulted in triggering slow-adapting Type II receptors (Beceren et al., 2013).

The augmenting effects of subthreshold vibratory noise stimulation may not be restricted to the actual body location of vibratory stimulation but can radiate to other locations as well. In stroke patients, for example, Enders and colleagues reported augmented fingertip sensitivity during stimulation at the wrist and dorsal skin surface of the paretic hand (Enders et al., 2013). Remarkably, fine motor dexterity of the paretic hand also seems to benefit from this remotely induced increased fingertip sensitivity (Seo et al., 2014). Suprathreshold vibration leads to reduced fingertip sensitivity, while the remote benefits of subthreshold stimulation seemingly do not depend on the exact location on the vibrated hand (Lakshminarayanan et al., 2015). The later finding led to the conclusion that any remote effects stem from central, possibly supraspinal origins. In a further study to follow-up the neurological basis of the remote effects of vibratory noise stimulation, Seo et al. reported increased somatosensory evoked potentials in the primary sensorimotor and premotor cortices during fingertip stimulation with simultaneous subthreshold wrist vibration of 60% of the vibrotactile threshold (Seo et al., 2015). Interestingly, it has also been suggested that improvement of sensorimotor performance during a visuomotor task via vibrotactile stimulation of the index finger is consistent with an increase in cortical motor spectral power and corticomuscular coherence (Trenado et al., 2014b).

Stochastic resonance can also influence motor learning. From the perspective of motor control, an error signal is exploited by the central nervous system to update its motor policy and reduce the discrepancy between observed and predicted errors (Diedrichsen et al., 2005). For instance, when playing darts, visual errors help us derive the best strategic change to eventually aim at the target. Improved signal quality can refine the internal state estimate and the accuracy of the error signal, therefore contributing to increased learning rates. As such, stochastic resonance can potentially facilitate movement learning processes that is based on integration of sensory inputs and sensory-motor coupling. Mendez-Balbuena et al. (2012) showed that an individually determined optimal level of mechanical noise significantly improves sensorimotor performance in a static force compensation task involving the index finger (Mendez-Balbuena et al., 2012). Effects of stochastic resonance were also studied in the context of sensorimotor performance during exercise induced muscle damage. Gleeson (2017) studied how application of mechanical vibrations to the biceps femoris muscle influences sensorimotor performance of target force replication (Gleeson, 2017). The study showed that subthreshold mechanical vibrations compensate the negative effects of muscle damage and suggested that more efficient means of delivering stochastic resonance (e.g., functional electrical stimulation) would be needed to improve the effectiveness to enhancing sensorimotor performance under adverse conditions of exercise stress.

### Visual Information

Vision is highly relevant for balance control as well (Figueiro et al., 2011). Stochastic resonance has been shown to enhance visual perception in humans by addition of pixel-noise to static scenes (Simonotto et al., 1997; Moss, 2004). Likewise, visual noise increased visual contrast detection sensitivity around threshold level and thereby improved pattern recognition and perception of ambiguous 3-D figures (Leopold et al., 2002; Sasaki et al., 2006). Also, it was demonstrated that adding background white pixel-noise to a random dot motion stimulus improved ability of participants to discriminate among motion’s direction (Treviño et al., 2016).

**FIGURE 2:**
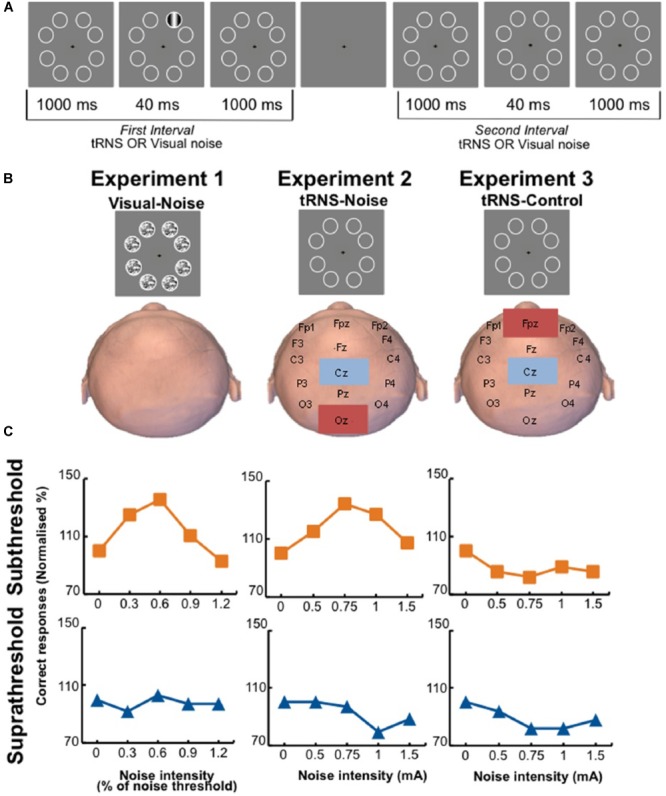
Participants were tested on a visual perception task in three different conditions. **(A)** Participants fixated on the center of the screen and a visual stimulus was randomly presented either in the first (shown here) or second interval. After the second interval, participants had to indicate which interval contained the stimulus. In the main experiment, the stimulus contrast was fixed to yield either 60% detection accuracy (subthreshold group) or 80% detection accuracy (suprathreshold group). **(B)** Representation of the three different experiments. In the tRNS–noise and tRNS–control experiments, no noise was presented on the screen. **(C)** Representative data of individual participants. The participants in the visual–noise (left) and tRNS–noise (middle) experiments show a peak in their detection performance when noise was added to a subthreshold stimulus (orange line), but not to a suprathreshold stimulus (blue line) (van der Groen and Wenderoth, 2016).

Remarkably, applying weak perturbations directly on the cortex also showed positive effects. Online low intensity transcranial magnetic stimulation facilitates detection of weak motion signals. In contrast, high intensities lead to impairment in detection. Thus, it was suggested that transcranial magnetic stimulation acts by adding noise to neuronal processing (Schwarzkopf et al., 2011). Recently, administration of transcranial random noise stimulation (100–640 Hz zero-mean Gaussian white noise with intensities ranging from 0 to 1.5 mA) to the occipital region of human participants resulted in detection accuracy of visual stimuli that followed an inverted U-shape function (Figure 2). The authors interpreted this effect as a stochastic resonance phenomenon (van der Groen and Wenderoth, 2016).

## Current Challenges, Recommendations and Future Perspectives

There is promise that combined stochastic resonance approaches may improve motor functions in older persons and in patients. There are, however, a number of challenges and broader open questions that prevent real applications from emerging.

Although targeting one sensory modality is already sufficient to demonstrate improvements on a certain performance outcome, the effect of combining more than one sensory modality has yet to be evaluated comprehensively within the context of falls and balance control. The potential interferences between modalities should be carefully addressed in appropriate experimental designs. Moreover, every individual may respond differently to the same stimulation. Theoretical work is needed to identify the relevant parameters that would allow for a personalized, online and optimal intervention. In the future, devices exploiting stochastic resonance should adopt smart approaches that will lead to restoration of impaired functions and that will be used intermittently.

While stochastic resonance is well documented theoretically, we lack clear understanding of its mechanisms and possible connections to other similar observed effects in biological (and non-biological) systems. The broadest possible definition of stochastic resonance is that it occurs when randomness has a positive role in a signal-processing context (McDonnell and Abbott, 2009). Initially, stochastic resonance was considered to be restricted to the case of periodic input signals. In fact, it now is widely used as an all-encompassing term, whether the input signal is a periodic sine wave, aperiodic or even chaotic. Depending on the input signal, the output performance takes the form of SNR (periodic) or mutual information (aperiodic) in function of noise level. Stochastic resonance reduces to vibrational resonance when the input signal is a high-frequency periodic force (Landa and McClintock, 2000; Deng et al., 2010) and to chaotic resonance when a system responds to a weak signal through the effects of chaotic activities (Nobukawa et al., 2017).

Over the course of evolution, the brain learned to integrate the effects of gravity – that cannot be removed – to optimize motor actions (Crevecoeur et al., 2009; White, 2015; Rousseau et al., 2016). Similarly, noise is ubiquitous at all levels, from cellular to perceptual and motor, and it is virtually impossible to remove it completely (Faisal et al., 2008). Motor strategies need to be set in ways that make use of the inherent noise to obtain an optimal response.

The concept of noise is closely related to the one of variability. Interestingly, the theoretical model of optimal movement variability (Stergiou and Decker, 2011) is based on the complementary concepts of complexity and predictability. The optimal state of a biological system may be characterized by chaotic temporal variations in the steady state output that correspond to maximal complexity (Lipsitz and Goldberger, 1992). Any deviation from healthy state, that may be induced by senescence or disease, causes a loss in complexity. Similarly to stochastic resonance, this effect has been observed in a very broad panel of contexts. In addition, a lack of practice results in high disorder (randomness or no predictability) and excessive practice leads to high order (periodic signal or maximal predictability). A system has the propensity to adapt best to external disturbances at an intermediate state of predictability. The human body behaves as a non-linear dynamical system and exhibit a complex structure associated to an infinite repertoire of behaviors at different time scales. A decrease of complexity of a system results from either a reduction in the number of structural components or an alteration in the coupling function between these components. For instance, the upper limb can become rigid with senescence, hence suppressing degrees of freedom and, consequently, reducing its complexity. A holistic approach to study these mechanisms requires to associate specific measurements to these two concepts. The Hurst exponent is well suited to reflect predictability and the Minkowski fractal dimension provides good measurability of the “apparent rugosity” of fractals and reflects complexity. These two indexes are correlated and converge to the same value if measurement time is set to infinity. However, they yield complementary information in more realistic contexts such as in gait analysis (Dierick et al., 2017).

The measure of performance should not be restricted to signal detection or some SNR. Instead, it is more sensible to asses variations in *functions* with changing noise level. Stochastic resonance would then predict that, for some non-zero noise, the function will work optimally. Similarly, optimal movement variability would predict that for some complexity/predictability, the function will be optimal as well. Theoretical questions remain open as to whether these two optimum are the same and how stochastic resonance and optimal movement variability relate to each other, possibly in the context of system stability (Boulanger et al., 2018).

Successful stochastic resonance studies mentioned in previous sections underline the potential to develop field prototypes. McDonnell and Abbott point out that “*… if it can be established that SR plays an important role in the encoding and processing of information in the brain, and that it somehow provides part of the brain’s superior performance to computers and artificial intelligence in some areas, then using this knowledge in engineering systems may revolutionize the way we design computers, sensors, and communications systems.*” (McDonnell and Abbott, 2009). A compromise between technological factors (e.g., footprint, power consumption), device versatility and end-user acceptability must be sought. A few projects dedicated to improving balance specifically or use vibrations with stochastic resonance in an attempt to improve some function have been conducted. For instance, two emblematic projects have demonstrated the use of mechanical vibrations to improve balance (Balancing Act) and writing (ARC Pen). Subjects with retinal disorder and impaired vision also showed enhancement of letter recognition at the same level as the one observed in normal sighted individuals when tested with visual prosthesis using stochastic resonance (Itzcovich et al., 2017). Nevertheless, going from a prototype to a clinically validated product is a long-lasting story that demands involvement of patients and increasing awareness about the safety and efficiency of the developed technology. Furthermore, in a world of connected smart technologies, stimulation delivery schedules should be dynamically optimized in function of well-defined behavioral factors and provide regular meaningful feedback reports to the patient. This last point is central as it ensures that the patient remains convinced of the usefulness of the technology.

## Author Contributions

All authors contributed to writing of this manuscript and conceptualized the review. OW conceived and designed the manuscript, conducted the literature search, interpreted the data for the work, and drafted the manuscript. JB and CT revised the work critically for important intellectual content. LJ and NG contributed to designing of the manuscript, interpreted the data and helped in editing the written manuscript.

## Conflict of Interest Statement

The authors declare that the research was conducted in the absence of any commercial or financial relationships that could be construed as a potential conflict of interest.
